# Expanding the mutation and phenotype spectrum of *MYH3*-associated skeletal disorders

**DOI:** 10.1038/s41525-021-00273-x

**Published:** 2022-02-15

**Authors:** Sen Zhao, Yuanqiang Zhang, Sigrun Hallgrimsdottir, Yuzhi Zuo, Xiaoxin Li, Dominyka Batkovskyte, Sen Liu, Hillevi Lindelöf, Shengru Wang, Anna Hammarsjö, Yang Yang, Yongyu Ye, Lianlei Wang, Zihui Yan, Jiachen Lin, Chenxi Yu, Zefu Chen, Yuchen Niu, Huizi Wang, Zhi Zhao, Pengfei Liu, Guixing Qiu, Jennifer E. Posey, Zhihong Wu, James R. Lupski, Ieva Micule, Britt-Marie Anderlid, Ulrika Voss, Dennis Sulander, Ekaterina Kuchinskaya, Ann Nordgren, Ola Nilsson, Deciphering Disorders Involving Scoliosis and COmorbidities (DISCO) study, Terry Jianguo Zhang, Giedre Grigelioniene, Nan Wu

**Affiliations:** 1 Department of Orthopedic Surgery, State Key Laboratory of Complex Severe and Rare Diseases, Peking Union Medical College Hospital, Peking Union Medical College and Chinese Academy of Medical Sciences, Beijing, 100730 China; 2 Beijing Key Laboratory for Genetic Research of Skeletal Deformity, Beijing, 100730 China; 3 Department of Orthopaedic Surgery, Qilu Hospital, Cheeloo College of Medicine, Shandong University, Jinan, Shandong 250012 China; 4 Division of Pediatric Endocrinology and Center for Molecular Medicine, Department of Women’s and Children’s Health, Karolinska Institutet and University Hospital, Stockholm, Sweden; 5 Key laboratory of big data for spinal deformities, Chinese Academy of Medical Sciences, Beijing, China; 6 Department of Molecular Medicine and Surgery, Karolinska Institutet, Stockholm, Sweden; 7 Department of Clinical Genetics, Karolinska University Hospital, Stockholm, Sweden; 8 Department of Joint Surgery, First Affiliated Hospital of Sun Yat-sen University, Guangzhou, 510080 China; 9 Department of Molecular and Human Genetics, Baylor College of Medicine, Houston, TX 77030 USA; 10 Baylor Genetics, Houston, TX 77021 USA; 11 Departments of Pediatrics, Texas Children’s Hospital and Baylor College of Medicine, Houston, TX 77030 USA; 12 Texas Children’s Hospital, Houston, TX 77030 USA; 13 Human Genome Sequencing Center, Baylor College of Medicine, Houston, TX 77030 USA; 14 Clinic of Medical Genetics and Prenatal Diagnostics, Children’s Clinical University Hospital, Vienibas gatve 45, Riga, LV-1004 Latvia; 15 Department of Pediatric Radiology, Karolinska University Hospital, Stockholm, Sweden; 16 Department of Clinical Genetics and Department of Clinical and Experimental Medicine, Linköping University, Linköping, Sweden; 17 School of Medical Sciences, Örebro University and Department of Pediatrics, Örebro University Hospital, Örebro, Sweden

**Keywords:** Disease genetics, Medical genetics

## Abstract

Pathogenic variants in *MYH3* cause distal arthrogryposis type 2A and type 2B3 as well as contractures, pterygia and spondylocarpotarsal fusion syndromes types 1A and 1B. These disorders are ultra-rare and their natural course and phenotypic variability are not well described. In this study, we summarize the clinical features and genetic findings of 17 patients from 10 unrelated families with vertebral malformations caused by dominant or recessive pathogenic variants in *MYH3*. Twelve novel pathogenic variants in *MYH3* (NM_002470.4) were identified: three of them were *de novo* or inherited in autosomal dominant way and nine were inherited in autosomal recessive way. The patients had vertebral segmentation anomalies accompanied with variable joint contractures, short stature and dysmorphic facial features. There was a significant phenotypic overlap between dominant and recessive *MYH3*-associated conditions regarding the degree of short stature as well as the number of vertebral fusions. All monoallelic variants caused significantly decreased SMAD3 phosphorylation, which is consistent with the previously proposed pathogenic mechanism of impaired canonical TGF-β signaling. Most of the biallelic variants were predicted to be protein-truncating, while one missense variant c.4244T>G,p.(Leu1415Arg), which was inherited in an autosomal recessive way, was found to alter the phosphorylation level of p38, suggesting an inhibition of the non-canonical pathway of TGF-β signaling. In conclusion, the identification of 12 novel pathogenic variants and overlapping phenotypes in 17 affected individuals from 10 unrelated families expands the mutation and phenotype spectrum of *MYH3*-associated skeletal disorders. We show that disturbances of canonical or non-canonical TGF-β signaling pathways are involved in pathogenesis of *MYH3*-associated skeletal fusion (MASF) syndrome.

## Introduction

*Myosin heavy chain 3* (*MYH3*) encodes the heavy chain of embryonic myosin, a muscle protein composed of a globular motor domain attached to a long coiled-coil rod domain by a short neck and a hinge region. Embryonic myosin exists as a dimer in which the tail domains are intertwined^1^. Hundreds of such dimers assemble with other proteins to form the sarcomere, the subcellular contractile apparatus of skeletal and cardiac muscle cells. *MYH3* is highly expressed during embryonic and fetal development, from gestational week 6 to week 24, and continues to be expressed postnatally at both mRNA and protein levels in skeletal and heart muscle and at the mRNA level in several other tissues (www.proteinatlas.org)^2^.

Monoallelic pathogenic variants in *MYH3* cause distal arthrogryposis (DA) syndromes, including arthrogryposis, distal, type 2A (DA2A, Freeman–Sheldon syndrome, MIM#193700) and arthrogryposis, distal, type 2B3 (DA2B3, Sheldon–Hall syndrome, MIM#618436)^3^. The phenotypes of DA2A and DA2B3 are variable but both are characterized by contractures of proximal and distal joints^4^. In addition to DA, heterozygous pathogenic variants in *MYH3* have been reported in contractures, pterygia and spondylocarpotarsal fusion syndrome 1A (CPSFSIA, MIM#178110), which is characterized not only by joint contractures, but also by multiple vertebral, carpal and tarsal fusions^5,6^. It has been proposed that pathogenic variants in *MYH3* inhibit TGF-β signaling, which is essential for the normal muscle function. The dysfunction of small muscles that attach at the distal neural arches of the spine is thought to lead to vertebral fusions^5^.

Recently, biallelic nonsense variants and splicing defects in *MYH3* were reported in contractures, pterygia and spondylocarpotarsal fusion syndrome 1B (CPSFSIB, MIM#618469)^7^. In total there are 29 individuals reported with contractures, pterygia and spondylocarpotarsal fusion syndrome 1A and 10 individuals with 1B^5,7–13^. Thus very little is known regarding the phenotype and mutation spectrum of CPSFSIA and CPSFSIB as well as their natural clinical course.

In this study we extend the phenotypic spectrum of *MYH3*-associated disorders reporting additional 17 affected individuals from 10 unrelated families with vertebral fusions, arthrogryposis and multiple pterygia. This report adds 12 novel pathogenic variants in *MYH3* and provides experimental evidence supporting pathogenicity of the missense variants in our patients using functional studies in HEK293T cells.

## Results

### Recruitment of patients with pathogenic *MYH3* variants

**Fig. 1:**
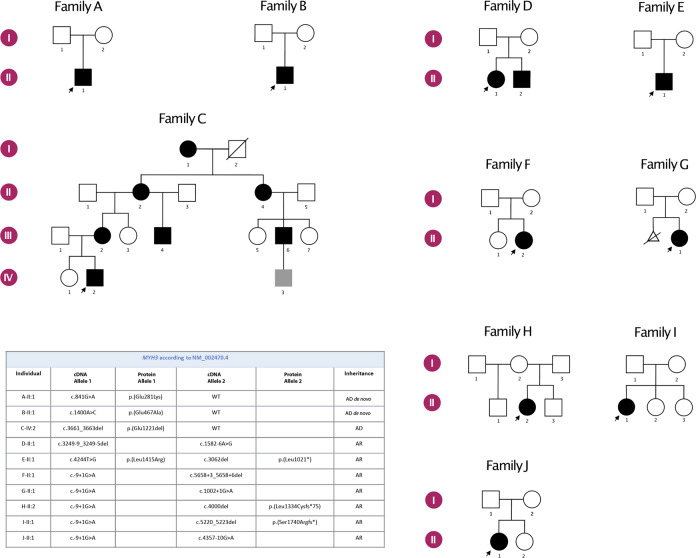
Pedigrees of the participating families and the pathogenic *MYH3* variants detected in the study. In family A, B and C the affected individuals have dominant *MYH3*-associated skeletal fusion syndrome, in families D, E, F, G, H, I and J the affected individuals have recessive *MYH3*-associated skeletal fusion syndrome. The pathogenic variants are summarized in the table below the pedigrees.

**Fig. 2:**
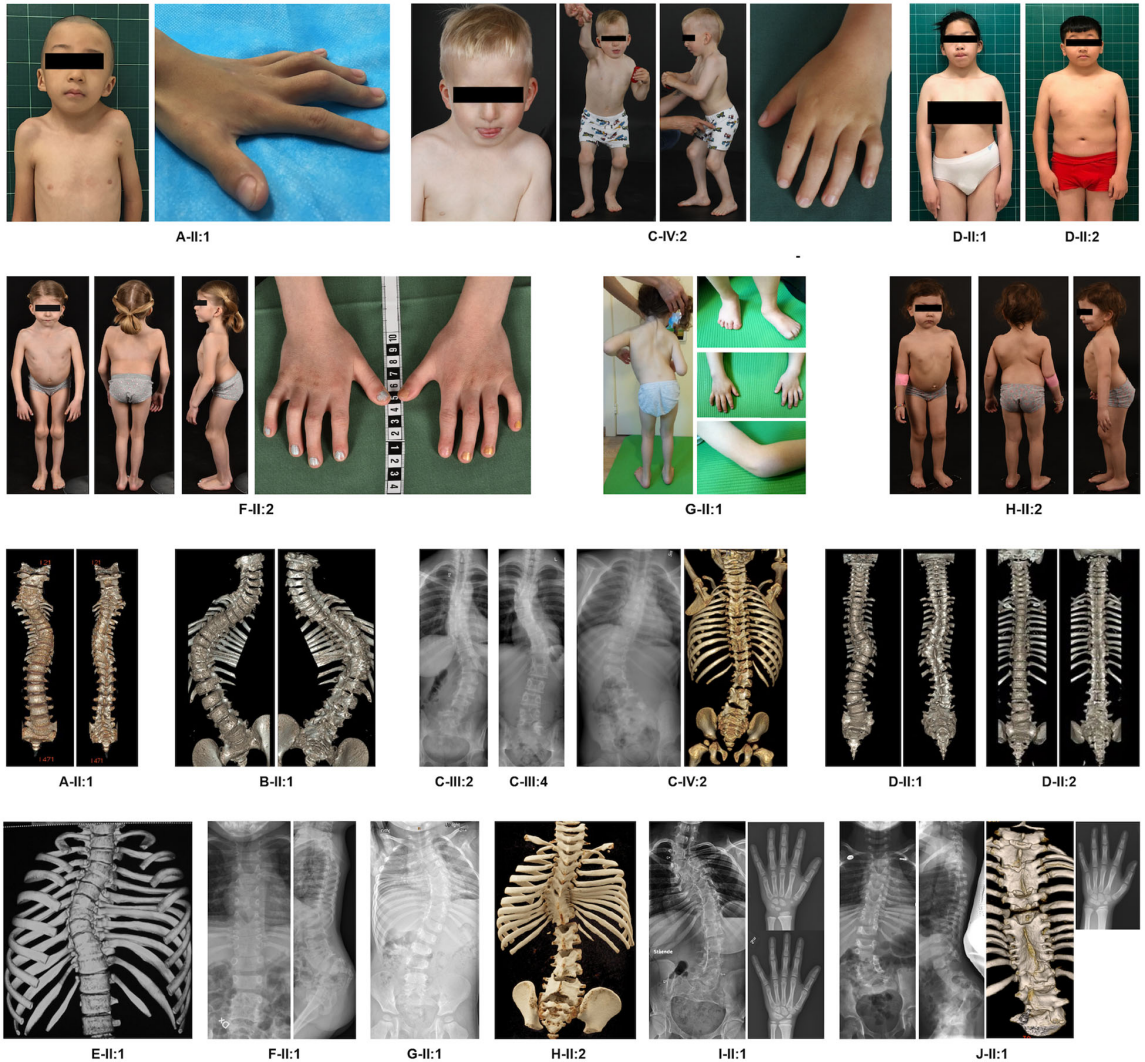
Phenotypic and radiographic features of the patients with pathogenic *MYH3* variants. Please note the following features: A-II:1 has small mouth with dowslanting corners, low-set ears, prominent nasal bridge and camptodactyly. C-IV:2 has small mouth, broad chest, increased distance between the nipples, enlarged knee joints with pterygia, flat feet and camptodactyly. D-II:1 has small mouth with downslanting corners, small low set ears, prominent philtrum and increased distance between the nipples. D-II:2 has small mouth with downslanting corners, small low set ears, prominent philtrum and ptosis. F-II:2 has broad chest, scoliosis, enlarged knee joints, elbow and knee contractures and bilateral clinodactyly of the 5th finger. G-II:1 has scoliosis and pterygia. H-II:2 has broad chest, scoliosis and mild pterygia of the knee. Radiographic features: A-II:1 has multiple cervical and thoracic vertebral fusions, rudimentary disc spaces and scoliosis. B-II:1 has multiple, mainly thoracic, vertebral fusions with rudimentary disc spaces, rib crowding and scoliosis. C-III:2 and C-III:4 both have moderate scoliosis and a few thoracic and lumbar/lumbosacral fusions. C-IV:2 has multiple posterior fusions of the cervical, thoracic and lumbar spine and scoliosis. D-II:1 has several thoracic and lumbosacral fusions with a distinct curve due to asymmetric T9 vertebral body. D-II:2 has some posterior vertebral fusions, mostly thoracic, without scoliosis. E-II:1 has multiple thoracic vertebral fusions, mild rib crowding and scoliosis. F-II:2 has multiple thoracic and lumbar vertebral fusions with rudimentary disc spaces, rib crowding and marked lumbosacral lordosis without scoliosis. G-II:1 has multiple thoracic vertebral fusions, rib crowding and scoliosis. H-II:2 has multiple thoracic and lumbar/lumbosacral posterior fusions, rib crowding and scoliosis. I-II:1 has multiple thoracic and lumbar vertebral fusions, rib crowding and scoliosis. Hand radiograms of I-II:1 at 5 and 8 years of age show development of lunotriquetral fusion. J-II:1 has multiple thoracic and lumbar vertebral fusions, rib crowding and scoliosis at 4 years 4 months of age. Hand radiogram shows lunotriquetral fusion.

We recruited ten families with congenital vertebral malformations and pathogenic variants in *MYH3*. Families A, B, D and E are of Chinese origin while families C, F, G, H, I and J are of European descent (Fig. 1). The phenotypic features, radiographic findings and the identified pathogenic variants are summarized in Figs. 1 and 2 and listed in Table 1. Information on the variant frequency, evolutionary conservation and *in silico* analyses is summarized in Table 2.

**Table 1:** Summary of clinical and radiographic features of the participants.

Families	A		B	C							D		E	F	G	H	I	J
	II:1		II:1	I:1	II:2	II:4	III:2	III:4	III:6	IV:2	II:1	II:2	II:1	II:2	II:1	II:2	II:1	II:1
Sex	M		M	F	F	F	F	M	M	M	F	M	M	F	F	F	F	F
Origin	China		China	Sweden	Sweden	Sweden	Sweden	Sweden	Sweden	Sweden	China	China	China	Sweden	Latvia	Sweden	Sweden	Sweden
Age in years	6		10	85	58	61	37	26	38	9	12	10	12	8	3	4	18	21
Skeleton, joints and limbs																		
Scoliosis	+		+	+	+	+	+	+	–	+	+	+	+	+	+	+	+	+
Vertebral fusion and malformations	C3-7, T3-4, T5-9, T9-11		T3-6, T7-8, T9 11, L4-S1	Self-reported fusion no radiograms available	L4-S1	Self-reported fusion no radiograms available	T4-5, T9-10, L4-S1	T3-5, T7-8, T10-11, T12-L1, L4-S1	NA	C6-7, T3-5, T6-7, T8-10, L1-2, L3-4	T5-6, T8 10, T9 hemi-vertebra, L5-S1	T5-6, T8-10, L4-S1	T3-4, T6-8, T9-11	C7-T2, T8-12, T5-6, L2-5, S1-2	T2-4, T6-8, T10-12 butterfly vertebra	T5-11, T12-L1, L2-3, L4-S1	T1-2, T3-5, T6-8, T9-11, L1-4	T1-2, T3-4, T6-11, L1-2, L3-5
Short neck	+		–	+	+	NA	+	–	+	+	–	–	–	–	+	–	+	+
Neck webbing	+		–	–	–	NA	+	–	+	+	+	+	+	+	+	+	+	-
Joint contractures	–		–	–	–	–	–	–	+	+	–	+	+	+	+	+	+	+
Camptodactyly	+		–	–	–	–	–	–	NA	+	–	+	–	+	NA	–	+	+
Joint pterygium	–		–	–	–	–	–	–	–	+	–	–	–	+	NA	+	+	-
Carpal/tarsal fusion	NA		NA	NA	NA	NA	NA	NA	NA	–	–	–	NA	–	–	–	–	+
Clubbed feet	–		–	–	–	–	–	–	–	+	–	–	–	–	–	–	–	-
Facial features																		
Small mouth	–		NA	–	–	NA	–	–	NA	–	–	–	–	+	+	+	–	+
Downslanting palpebral fissures	+		NA	–	–	NA	+	–	NA	+	–	–	+	+	+	–	–	+
Ptosis	–		NA	–	–	NA	–	–	NA	–	–	+	–	–	–	–	–	-
Prominent nasal bridge	+		NA	+	+	NA	+	+	NA	+	+	–	+	+	–	–	–	+
Low-set, posteriorly rotated ears	+		NA	+	+	NA	+	+	NA	+	+	–	+	+	–	+	–	-
Prominent philtrum	+		NA	+	+	NA	+	+	NA	+	+	–	+	+	+	+	–	-
Cleft palate	+		NA	–	–	–	–	–	–	–	–	–	NA	–	–	–	–	-
Other symptoms/features																		
Height *Z*-score age	−3.2 10 y	−2.6 10 y		−1.3 adult*	−1.1 adult*	−1.3 adult	−1.3 adult	−1.0 adult	−0.7 adult	−2.6 8 y	−2.6 12 y	NA	−1.4 12 y	−4.4 7 y	−1.3 3 y	−1.9 4 y	–1,9 adult	−2,9 adult
Other	Inguinal hernia, low posterior hairline		Gait disorder	*Hallux valgus*, right ear hypoplasia, external auditory canal atresia, hearing loss	*Hallux valgus*, decreased elbow extension, joint pain and stiffness	–	*Hallux valgus*. Treated with GH for short stature, minimal effect	–	Treated with GH for short stature, moderate effect	Bilateral cryptorchidism, mild learning disability	Low posterior hairline	–	Low posterior hairline	–	Hip dysplasia, retrognathia		Short neck, bilateral single palmar creases, bicuspid aorta valve, treated with GH for short stature, moderate effect	Short neck, unilateral single palmar crease, smooth filtrum, slight facial asymmetry

Adult heights were calculated using final heights from children auxology tables for 18 yo https://www.who.int/childgrowth/standards/en/.

*+* feature is present, *–* feature is absent, *NA* not available, *M* male, *F* female, *y* years, * self-reported, *GH* growth hormone.

**Table 2:** Sequence variants in *MYH3*, their frequencies in gnomAD and bioinformatic scores.

Family	Inheritance	Nucleotide change^#^	Predicted amino acid change	gnomAD*	PolyPhen-2	SIFT	Phylo-P	Affecting splice^a^	CADD
A	AD	c.841G>A	p.(Glu281Lys)	Not reported	0.995	Deleterious	6.02	No	32
B	AD	c.1400A>C	p.(Glu467Ala)	Not reported	1.000	Deleterious	4.73	No	28
C	AD	c.3661_3663delGAG	p.(Glu1221del)	Not reported	NA	NA	NA	No	NA
D	AR	c.3249–9_3249–5delTCTTC	–	Not reported	NA	NA	NA	Yes	NA
		c.1582-6A>G	–	0.00058	NA	NA	−0.36	Yes	21
E	AR	c.3062delT	p.(Leu1021*)	Not reported	NA	NA	5.05	No	NA
		c.4244T>G	p.(Leu1415Arg)	Not reported	1.000	Deleterious	5.05	No	30
F	AR	c.–9+1G>A	–	0.01064	NA	NA	2.87	Yes	33
		c.5658+3_5658+6del	–	Not reported	NA	NA	NA	Yes	16
G	AR	c.–9+1G>A	–	0.01064	NA	NA	2.87	Yes	33
		c.1002+1G>A	–	0.00004	NA	NA	6.02	Yes	34
H	AR	c.–9+1G>A	–	0.01064	NA	NA	2.87	Yes	33
		c.4000del	p.(Leu1334Cysfs*75)	Not reported	NA	NA	NA	No	33
I	AR	c.–9+1G>A	–	0.01064	NA	NA	2.87	Yes	33
		c.5220_5223del	p.(Ser1740Argfs*)	Not reported	NA	NA	NA	No	NA
J	AR	c.–9+1G>A	–	0.01064	NA	NA	2.87	Yes	33
		c.4357-10G>A	–	0.00008	NA	NA	−0.3	Yes	12

*NA* not available.

^#^NM_002470.4 (*MYH3*), *dataset v2.1.1 based on highest MAF in population, ^a^affecting splice according to Alamut visual 2.14.

### Autosomal dominantly inherited or *de novoMYH3* variants and phenotype of the patients

In families A, B and C, *de novo* or dominantly inherited heterozygous variants in *MYH3* were identified. All these variants are novel.

**Fig. 3:**
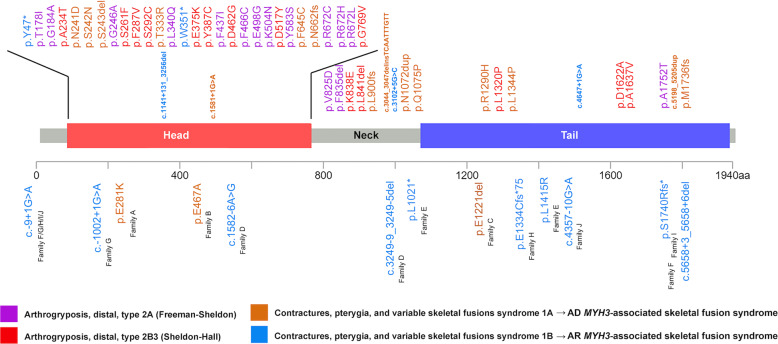
Schematic representation of MYH3 variants in published literature (upper part of the figure) and in this study (lower part of the figure). Different colors annotate variants associated with different forms of *MYH3*-associated conditions.

Proband A-II:1 from family A is a 7-year-old Chinese boy born at GA 37 + 3. Skeletal survey showed multiple vertebral fusions, short neck with pterygium, increased distance between nipples, *pectus excavatum* and moderate short stature (Table 1). His facial features include downslanting palpebral fissures, high nasal bridge, cleft palate, low set ears and camptodactyly of the left hand. Cognitive and motor development are normal. He is heterozygous for a *de novo* variant in *MYH3*: c.841G>A, p.(Glu281Lys), which is located in the head domain of MYH3 (Table 1, Fig. 3).

The proband from family B, B-II:1, is a 10-year-old male born at GA 38 + 4. He presented with *torticollis* at birth. Skeletal examinations showed congenital scoliosis, multiple vertebral fusions and moderate short stature (Table 1 and Fig. 2). The gross motor development of the proband is mildly delayed and cognitive development is normal. This patient has a *de novo* heterozygous variant in *MYH3*: c.1400A>C, p.(Glu467Ala), also located in the head domain of MYH3 (Table 1, Fig. 3).

The proband (IV-2) in family C, C-IV:2, is a 9-year-old Caucasian male, born at gestational week 38 + 3 to non-consanguineous Swedish parents. He presented at birth with joint contractures and enlargement, popliteal pterygium, short neck, low set posteriorly rotated ears, which are different in size, *pterygium colli*, bilateral cryptorchidism and unilateral inguinal hernia. He developed scoliosis and spine radiographs revealed multiple vertebral fusion of the cervical, thoracic and lumbar spine (Fig. 2). Postnatally his height *z*-score decreased from −1.5 at birth to −2.9 at 6 years of age (Supplementary Fig. 1). He has late speech development and is undergoing developmental evaluation for mild learning disabilities. Family C includes seven affected individuals, all with short stature and vertebral anomalies, but otherwise variable features, summarized in Table 1. All affected members in this family are heterozygous for an in-frame deletion in *MYH3*: c.3661_3663delGAG, p.(Glu1221del) in exon 27, which is not present in their unaffected relatives (*n* = 7). Exon 27 encodes the neck domain of MYH3 (Table 1, Fig. 3).

### Autosomal recessively inherited *MYH3* variants and phenotype of the patients

In addition, seven pairs of compound heterozygous variants validated through parental sequencing were detected in two Chinese families, four Swedish families and one family from Latvia (Families D, E, F, G, H, I, J in Fig. 1). Nine of the recessive variants were novel, while c.–9+1G>A was found in 5 of our patients. Since the latter was previously reported by Cameron-Christie et al. and proven to be pathogenic in functional studies^7^ we did not pursue cDNA analyses. Molecular consequences of the novel splicing variants detected in families D, E, F and J are summarized in Fig. 5.

Family D has two affected siblings. The proband, D-II:1, is a 17-year-old female born at GA 38 + 5. She presented with congenital scoliosis, thoracic vertebral malformations and short stature. Her facial features include long nasal bridge, low-set, posteriorly rotated ears and prominent philtrum. She does not have joint contractures. D-II:2 is a 15-year-old male born at GA 39 + 2. He has thoracic vertebral fusions and contracture of the 5th proximal interphalangeal joint of left hand (Table 1, Fig. 2). Both siblings show moderate developmental delay. They are compound heterozygous for splicing variants in *MYH3*: c.3249-9_3249-5delTCTTC; c.1582-6A>G (Fig. 5).

The proband from family E, E-II:1, presents with multiple thoracic vertebral fusions, joint contractures, downslanting palpebral fissures, ptosis, long nose, low-set ears and wide philtrum (Table 1 and Fig. 2, for this individual only spinal CT is available for publication). He is compound heterozygous for two variants in *MYH3*: c.3062delT, p.(Leu1021*); c.4244T>G, p.(Leu1415Arg) (Fig. 5).

The proband in family F, F-II:2 is a 7 years 5 months old Caucasian girl born at GA 41 + 1. At birth, she presented with short extremities, broad chest with wide intermammillary distance, downslanting palpebral fissures, short neck with mild pterygium and reduced extension of the knees and fingers. Her skeletal survey shows multiple segmentation anomalies, reduced vertebral body and disc height, abnormally angulated and crowded ribs, anteriorly angled sacrum and subtle mesomelia of the upper extremities (Fig. 2). She suffers from severe progressive postnatal growth failure (Supplementary Fig. 1). She received continuous physical therapy from birth with improvement in mobility and has not needed any orthopedic surgical corrections. Cognitive development is normal and gross motor development mildly delayed. During her first years of life she experienced mild feeding problems, recurrent middle ear infections as well as recurrent episodes of obstructive bronchitis. She is compound heterozygous for two splicing variants in *MYH3*: c.–9+1G>A; c.5658+3_5658+6del (Fig. 5).

The proband in family G, G-II:1, was born to non-consanguineous parents of Caucasian origin from a second IVF pregnancy. IVF was due to ovarian cysts in the mother. She was born at GA 39 + 2 with Apgar 8, 9 at 1 and 5 min. Newborn physical examination revealed bilateral single transverse palmar creases, sacral skin fold with slight hypertrichosis and left sided mild hip dysplasia. MRI at 2 weeks of age showed scoliosis at the thoracic portion of the spine, fused arches in vertebral blocks T2-T4, T6-T8 and T10-T12 and butterfly vertebrae at T10 and T12. Her psychomotor development is normal. She has short neck, 10° contractures of elbows and knees, micrognathia, left palpebral ptosis and hypoplastic *alae nasi*. At 28 months she had hypermetropia +3.5D. She is compound heterozygous for two splice variants: *MYH3*: c.–9+1G>A; c.1002+1G>A (Fig. 5).

The proband in family H, H-II:2, is a 4-year-old female, born to unrelated parents of Caucasian origin at GA of 40 + 6. Her family history is unremarkable except for mild scoliosis in her mother. Her parents noticed spine deformity at 3 months of age and she was diagnosed with multiple vertebral fusions at age of 3 years. She has scoliosis, a relatively wide thorax, contractures of the knees and hips, normal psychological development and presents with mild short stature at 4 years. The patient is compound heterozygous for variants *MYH3*: c.–9+1G>A and c.4000del, p.(Leu1334Cysfs*75) (data not shown).

The proband from family I, I-II:1, is an 18-year-old female, born to unrelated parents of Swedish origin at GA 41 + 5 after induction of delivery and vacuum extraction. Prenatal ultrasound examinations were normal. Apgar was 9, 10, 10 at 1, 5 and 10 min respectively. Delivery was complicated by a clavicular fracture. Short stature and scoliosis came to medical attention at 15 months. From 3 years of age, she suffered from a few episodes of painful, swollen knee joints. Juvenile idiopathic rheumatoid arthritis was suspected and the patient received various treatments, including methotrexate from 3 years and 8 months to 6 years of age. A mild 5-degree extension defect of one knee joint, some limitations/stiffness in the flexion of the wrist joints and an inability to elevate the right shoulder beyond 160 degrees were noted. She was treated with growth hormone from 4 years and 8 months until 15 years of age. Her psychomotor development is normal. The patient has a short neck with pterygium, broad and relatively short chest, bilateral single palmar creases, scoliosis and extensive vertebral anomalies in the thoracic and lumbar region. The patient has a bicuspid aortic valve, which is also present in her father. Her father does not show any signs of skeletal dysplasia or joint contractures and we suspect that his heart malformation is due to another yet unidentified genetic reason. The patient is compound heterozygous for variants *MYH3*: c.–9+1G>A and c.5220_5223del, p.(Ser1740Argfs*2) (data not shown).

The proband in family J, J-II:1, is a 21-year-old female born at term to non-consanguineous Swedish parents. During the first year a congenital scoliosis became evident and spine radiographs revealed multiple vertebral fusions (Fig. 2). Further radiologic examinations showed a tethered cord and a combined surgery for tethered cord release and vertical expandible prosthetic titanium rib was performed at 4 years of age. She has a broad thorax and mild dysmorphic features including slight facial asymmetry (Table 1). Limited extension of the knees, hips, elbows and fingers became more evident with age. Length at birth was normal (*z*-score +0.2) but she showed successive growth failure and her final height is at *z*-score of −2.9 (Supplementary Fig. 1). During the first year she developed obstructive bronchitis that is still treated. Her motor and cognitive development is normal. She is compound heterozygous for two splicing variants in *MYH3*: c.-9+1G>A; c.4357-10G>A (Fig. 5).

### Significant phenotype variability and overlap among the patients with autosomal dominant and autosomal recessive *MYH3-*associated diseases

The affected individuals with dominant and recessive *MYH3*-associated conditions show variable degrees of vertebral fusions, short stature and dysmorphic features. We have collected available growth data from our patients’ medical records (*n* = 17) and calculated the height *z*-scores (Supplementary Fig. 1) using the WHO growth standards. For nine individuals in our study some retrospective growth data was available, for seven individuals we had only single height measurements and in one case no growth data was available. We depicted the height data from our patients comparing it with the height data from the patients reported in the literature^8,10–13^ (*n* = 17, Supplementary Fig. 1 and Supplementary Table 1). We find an overlap regarding the degrees of short stature in patients with autosomal dominant and recessive *MYH3*-associatedd diseases (Supplementary Fig. 1). In addition, the number of vertebral fusions was overlapping in our patients (Table 1). Height *z*-scores in our cohorts ranged from 0 to −4.4 indicating that pathogenic variants in *MYH3* lead to mild to moderate growth failure (Supplementary Fig. 1).

### Missense variants result in abnormal TGF-β signaling

**Fig. 4:**
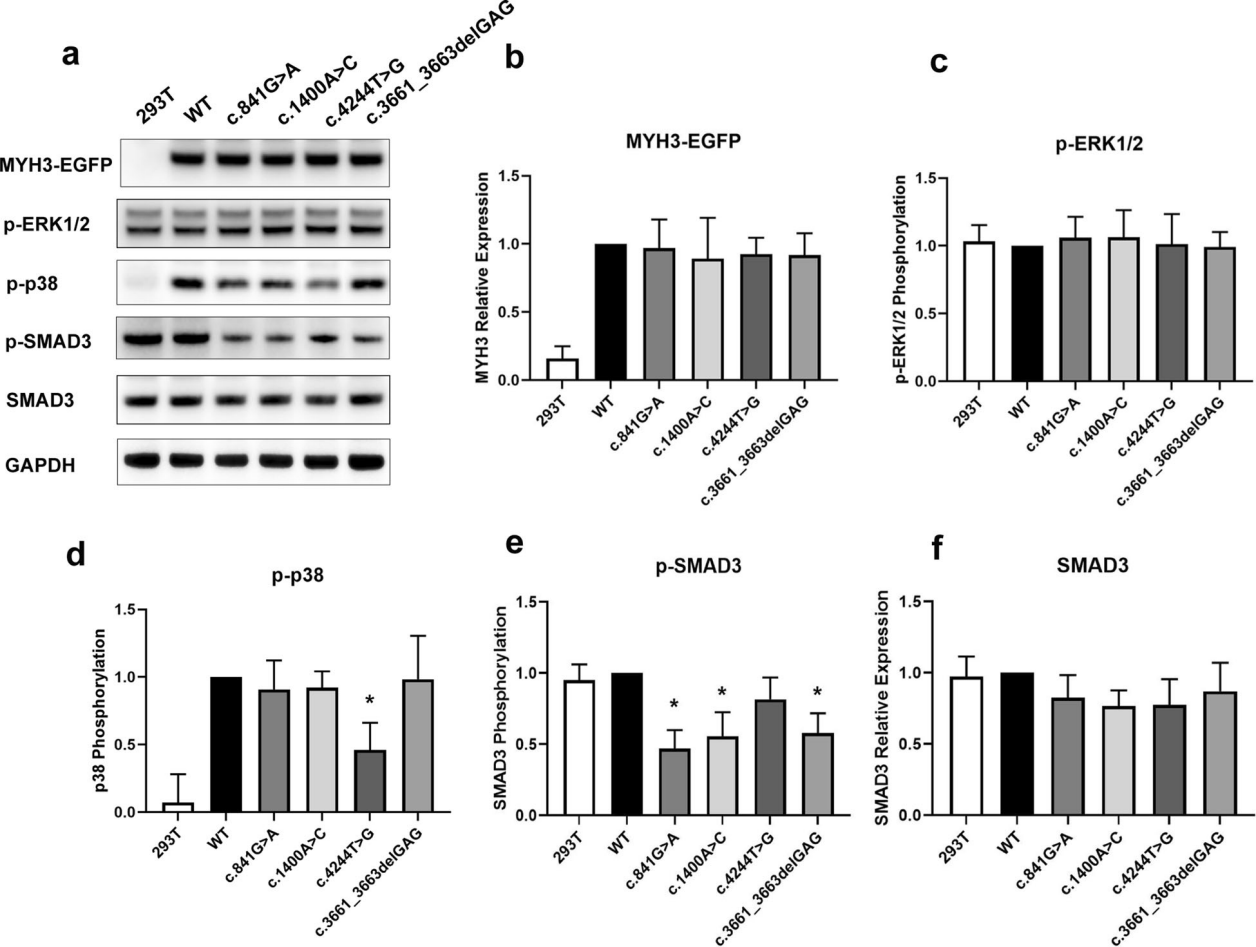
Expression of MYH3 and proteins in TGF-β/BMP signaling pathway (SMAD3 and phosphorylated SMAD3) in cells transfected with wild-type (WT) and mutant *MYH3* plasmid. **a** Western blot results of MYH3 expression and proteins in TGF-β/BMP signaling pathway in HEK 293T cells transfected with empty plasmid, WT and mutant *MYH3* plasmid. **b** Quantification of the MYH3 protein. **c**–**f** Quantification of TGF-β/BMP signaling pathways alterations on protein expression levels pERK1/2, p-p38, p-SMAD3, and SMAD3. c.841G>A, c.1400A>C and c.3661_3663delGAG variants lead to decreased stimulatory effect of MYH3 on SMAD3 phosphorylation, whereas c.4244T>G leads to decreased p-p38 expression. Western blot data was analyzed as ratios against samples transfected with the WT plasmid. The WT plasmid samples were set at a value of 1. The results are shown as the mean ± standard error of three independent experiments.

As protein-truncating variants in *MYH3* probably cause LoF of the protein, missense variants are more likely to have variable effects. Therefore, we examined the missense variants of unknown clinical significance found in this study using an over-expressing cell model. Using a *MYH3*-EGFP fusion plasmid we transfected constructs harboring the variants associated with the dominant disorder (c.841G>A, c.1400A>C, c.3661_3663delGAG) and with the recessive disorder (c.4244T>G) into HEK 293T cells. We found that none of the variants affected the expression of MYH3, SMAD3, or the phosphorylation levels of Erk1/2 (Fig. 4a, b, c, f). Phosphorylation levels of SMAD3 were significantly decreased in cells transfected with the dominantly inherited or *de novo* variants as compared with cells transfected with control wild-type plasmid (Fig. 4e, *P* = 0.0013, *P* = 0.0051 and *P* = 0.0075, respectively), suggesting an inhibition of the canonical TGF-β signaling pathway. In contrast, we found a decrease in p38 phosphorylation in HEK 293T cells transfected with the recessively inherited variant (c.4244T>G), indicating an inhibition of the non-canonical TGF-β signaling (Fig. 4d, *P* = 0.0051). These results indicate that different variants in *MYH3*-associated disorders may act through different mechanisms, i.e. interfere with the canonical or non-canonical TGF-β signaling, respectively.

### Abnormal splicing due to variants located outside the canonical splice sites

**Fig. 5:**
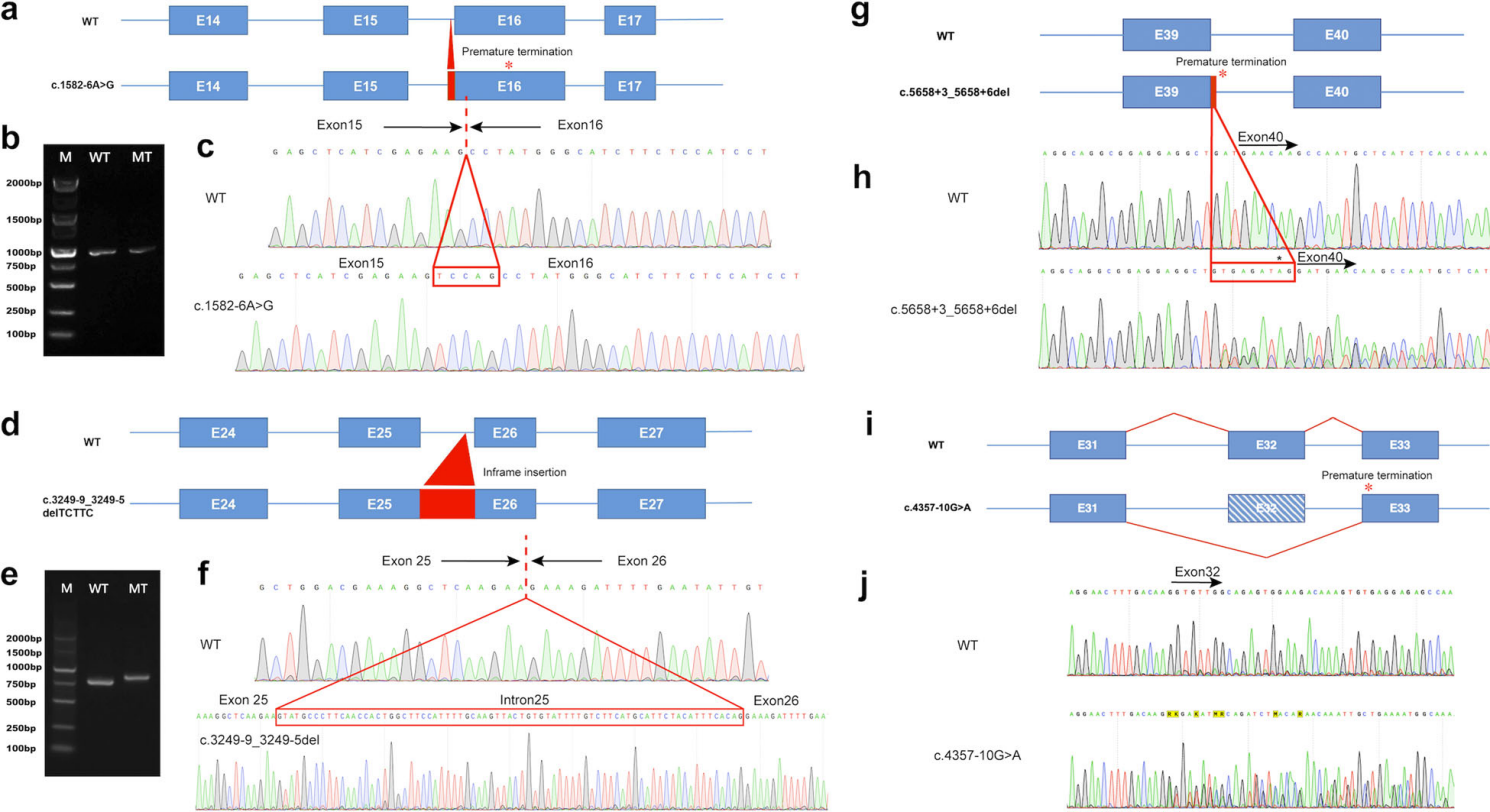
Analysis of the *MYH3* splicing abnormalities in families D, E, F, and J. **a** Schematic representation of the effect of c.1582−6A>G in Family D. **b** Agarose gel electrophoresis does not show difference in the product size because the length difference of the products is only 5 bp. **c** The c.1582-6A>G leads to an insertion of 5-bp of intronic sequence before exon 16, leading to a frameshift. **d** Schematic representation of c.3249-9_3249-5delTCTTC on molecular consequences on cDNA in family E. **e** Agarose gel electrophoresis illustrating the effect of the splicing variant c.3249-9_3249-5delTCTTC with a larger fragment size in the mutant allele. **f** Note that the pathogenic variant leads to retention of intron 25, and an in-frame insertion. **g** Schematic representation of c.5658+3_5658+6del molecular consequences on cDNA in family F. **h** Sanger sequencing of cDNA from the affected individual and her healthy parents shows that the c.5658+3_5658+6del leads to 9 bp intron retention between exon 39 and exon 40. *In silico* translation tool in *SIB ExPASy Bioinformatics Resources Portal* predicts a 9 bp inclusion, adding two amino acids followed by a termination codon annotated by * in the picture. **i** Schematic representation of c.4357-10G>A molecular consequences on cDNA in family J, showing that the variant leads to exon 32 skipping. **j** Sanger sequencing of cDNA from the affected individual shows end of exon 31 (single peaks in the chromatogram) and its boundaries to exon 32 and 33 respectively at the start of double peaks in chromatogram.

To confirm the predicted abnormal splicing caused by the variants located outside canonical splice sites, we performed a minigene assay for c.1582-6A>G and c.3249-9_3249-5delTCTTC. The c.1582−6A>G variant was shown to insert a 5-bp intronic sequence before exon 16, causing a frameshift and a premature stop codon after 55 amino acids (Fig. 5a, b, c). In contrast, c.3249-9_3249-5delTCTTC resulted in partial retention of intron 25 (Fig. 5d, e, f).

Sanger sequencing of cDNA from the proband in family F and her healthy parents showed that her variant c.5658+3_5658+6del led to a 9 bp intron retention between exon 39 and exon 40 (Fig. 5g, h). When applied to the *in silico* translation tool in *SIB ExPASy Bioinformatics Resources Portal*, this 9 bp inclusion is predicted to incorporate two additional amino acids followed by a termination codon as shown in Fig. 5h. Sanger sequencing of cDNA from the proband in family J, and her healthy parents showed that her variant c.4357-10G>A led to exon 32 skipping (Fig. 5i, j).

Notably, The c.–9+1G>A variant was detected in all Caucasian patients in *trans* with other splice or frameshift variants. This variant has been reported as pathogenic by previous functional studies^7^.

## Discussion

In this study, we report 12 novel pathogenic variants in *MYH3*, examine the functional consequences of disease associated variant alleles and summarize the clinical phenotypes of 17 affected individuals from 10 unrelated families. The affected individuals show variable manifestations of vertebral fusions, short stature and dysmorphic features. The clinical data indicate that there is a significant overlap in the degree of short stature as well as the number of fused vertebrae between our patients with autosomal dominant and autosomal recessive *MYH3*-associated disorders. These diagnoses are currently annotated as contractures, pterygia and spondylocarpotarsal fusion syndromes IA and IB (Supplementary Table 1, OMIM MIM#178110 and #618469). By noting this considerable clinical overlap, we suggest that these two Mendelian conditions represent variable expression of the same clinical entity and consequently should be termed *MYH3*-associated skeletal fusion (MASF) syndrome, with annotation of two different inheritance patterns. Even if the number of patients in this report is limited, our results showing the broad phenotypic variability between and within the families indicate that future studies are unlikely to find consistent and clear-cut phenotypic differences between autosomal and recessive MASF syndromes.

Until the recent identification of *MYH3* variants associated with contractures, pterygia and spondylocarpotarsal fusion syndromes 1A and 1B, most pathogenic variants in *MYH3* were associated with DA2A (Freeman–Sheldon syndrome, MIM #193700) and DA2B3 (Sheldon–Hall syndrome, MIM #601680), which are both characterized by limb contractures without vertebral anomalies^2,3,14,15^ as summarized in Supplementary Table 1. In some patients with DA2A, scoliosis and short stature have been described^2^, indicating presence of a phenotypic continuum between *MYH3*-associated syndromes with arthrogryposis with or without signs of skeletal fusions. DA2A and DA2B3 are associated with missense variants which are mostly located in the head and the neck domain of the myosin protein (Fig. 3). Consistent with the Cameron-Christie et al. study^7^ of contractures, pterygia and spondylocarpotarsal fusion syndrome IB, our results show that biallelic variants in *MYH3* causing recessive vertebral fusions are more likely LoF and might be protein-truncating or leading to nonsense mediated decay (NMD).

Cameron-Christie et al.^7^ and our study identified the c.–9+1G>A allele as a recurrent variant in European patients with contractures, pterygia and spondylocarpotarsal fusion syndromes 1B (AR MASF). This variant has a minor allele frequency of 0.01 in the Finnish population, 0.002 in the non-Finnish European population and is observed once in the African population (frequency according to gnomAD: https://gnomad.broadinstitute.org/variant/17-10559406-C-T). However, we did not observe this variant in any patient from the Chinese cohort or the East Asian population from gnomAD. It is possible that the position of the MYH3:c.−9+1G>A variant was not covered by the exome kits used in ES of the Chinese cohort, but the absence of it in East Asian population in gnomAD indicates that it might be very rare. This variant was present in all our Caucasian patients with AR MASF in compound heterozygous state with other splice site or frameshift variants. The high MAF of the c.–9+1G>A variant in the Finnish population seem to be contradicting with the rare prevalence of *MYH3*-associated diseases. A similar phenomenon has been observed in *TBX6*-associated congenital scoliosis, where a rare null *TBX6* variant in compound heterozygosity with a common haplotype causes congenital vertebral malformation^16,17^. It is possible that the c.–9+1G>A in homozygous state does not lead to phenotypic abnormalities, since having that high population carrier frequency the condition would have been reported as a genetic skeletal disorder commonly occurring in Finland. Taken together, we suggest that the hypomorphic variants should be explored when a highly pathogenic variant exists in *trans* in an affected individuals with features of MASF syndrome, particularly in populations that have a relatively high carrier rate of this variant, e.g. Finnish population.

TGF-β signaling is considered to be a crucial pathway for development and function of skeletal muscle and bone, including osteogenic or myogenic differentiation and regeneration^18,19^. Specifically, it has been shown to control proliferation, differentiation, apoptosis, migration, extracellular matrix (ECM) remodeling, immune functions and tumor invasion/metastasis^20^. In this study, we show that variants causing AD or AR forms of MASF act through different mechanisms. While variants causing the AD form of disease perturb canonical TGF-β signaling, pathogenic variants involved in AR form of disease either lead to truncated transcripts or interfere with the non-canonical TGF-β signaling. The canonical TGF-β signaling directly phosphorylates Smad3 to initiate signal transduction through the canonical cascades via activation of type I and type II serine/threonine kinase receptors^21,22^. In contrast, the non-canonical pathway mediates the activation of the mitogen-activated protein kinases (MAPKs) extracellular signal-related kinase (ERK), c-Jun N-terminal kinase (JNK) and p38^23,24^. Both canonical and non-canonical TGF-β signaling pathways are associated with musculoskeletal differentiation, migration and ECM remodeling^20^. The perturbation of canonical TGF-β signaling may confer more deleterious defects than non-canonical TGF-β signaling since the heterozygous variant c.4244T>G, p.(Leu1415Arg), which was inherited autosomal recessively in this study, did not lead to congenital vertebral malformations in the carrier parent. Similarly, functional studies in the Cameron-Christie et al. study^7^ demonstrated that the c.–9+1G>A variant resulted in a partial LoF of *MYH3*. Therefore, we hypothesize that the variants associated with the recessive forms of MASF are null (e.g., protein-truncating variants or variants leading to NMD) or hypomorphic (e.g., the c.–9+1G>A variant) in heterozygous state and therefore two affected alleles are necessary to lead to phenotypic abnormalities^25^. Further studies are needed to prove or discard this hypothesis, since the number of affected individuals in this report is relatively small.

In summary, we present both monoallelic and biallelic pathogenic variants in *MYH3* associated with heterogeneous and clinically overlapping features in patients with congenital vertebral malformations, providing further evidence for impaired TGF-β signaling in pathogenesis of *MYH3*-associated skeletal fusion syndrome.

## Methods

### Patients

Patients with disease-causing variants in *MYH3* were selected from a Chinese cohort of vertebral malformations under the framework of Deciphering disorders Involving Scoliosis and COmorbidities (DISCO) study (http://discostudy.org/) at Peking Union Medical College Hospital (PUMCH) (total cohort size *n* = 583) and from an European cohort of patients with congenital skeletal abnormalities studied at the Karolinska University Hospital (total cohort size *n* = 277). The study was approved by the Ethics Committee of Peking Union Medical College Hospital (JS-908) and by Regional Ethical Review Board for Karolinska University Hospital (protocol numbers are 2014/983-31/1 and 2012/2106-31/4). Informed consent was obtained from all affected patients or their parents/legal guardians. Clinical data were collected from the patients’ records by their referring physicians. The *z*-scores of patients length/height were calculated using WHO Child growth standards (https://www.who.int/childgrowth/standards/en/)^26^.

### Sequencing and in-house genomic databases

For the Chinese cohort, exome sequencing (ES) was performed as previously described^27^. For the Swedish cohort, clinical whole-genome sequencing (WGS) was performed as previously described including calling for SNVs, CNVs, and SVs^28^. As a part of a standard clinical genome analysis we look at CNVs and SVs in all our patients with suspected genetic syndromes^29^. For the Swedish cohort, we have also looked for clinically significant CNVs and SVs in the patients who underwent WGS, but no significant abnormalities were detected. DNA of the proband in family J, J-II:1, who underwent clinical ES in 2013, also was examined using aCGH as previously described^30^ with normal result. *MYH3* (NM_002470.4) variants were queried from the ES or WGS data and segregation analysis was performed in the families using Sanger sequencing. The variants were prioritized according to expected disease-causing potential, their presence in coding exons ±20 base pairs intronic sequence and a max minor allele frequency of 0.005 according to public (1000 G, dbSNP, ExAC/gnomAD, and ClinVar) and two country-specific in-house databases. An in-house database consisting of ES data from 4246 unrelated Chinese individuals without apparent scoliosis was utilized to determine the frequency of candidate variants in the Chinese Han population. The Swedish population reference database consists of whole genomes of 5015 individuals. The sample of patient J in the Swedish cohort was analysed using ES and conventional Sanger sequencing for the European hot spot variant *MYH3*: c.–9+1G>A, since it was not covered by ES (primers available at request).

### *MYH3* plasmid site-directed mutagenesis

The wild-type full-length cDNA of *MYH3* amplified from the peripheral blood RNA using primer 5'TGGGAGGTCTATATAAGCAGAG3' (forward) and primer 5' CGTCGCCGTCCAGCTCGACCAG3' (reverse) and cloned into the *Xho*I and *Hind*III sites of the pEGFP expression vector (pcDNA3.1-EGFP). Inverse PCR-based site-directed mutagenesis (TOYOBO, Japan) was used to construct the *MYH3* missense variants.

### RT-PCR and cDNA sequencing

RT-PCR and RNA sequencing were performed in Swedish families F and J to explore whether their intronic variants (*MYH3*: c.5658+3_5658+6del and c.4357-10G>A) lead to abnormal mRNA splicing. RNA was extracted from white blood cells using the RNeasy Mini Kit (QIAGEN, Hilden, Germany). First-strand cDNA was synthesized with M-MLV Reverse Transcriptase (Life Technologies, Carlsbad, CA, USA). cDNA was amplified, examined by gel electrophoresis and Sanger sequenced according to standard protocols. The sequences of the novel transcripts were analyzed using the *in silico* translation tool in *SIB ExPASy Bioinformatics Resources Portal*^31^.

### Western blot

*MYH3* plasmids were transfected into human embryonic kidney HEK293T cells using the Lipofectamine™ 3000 Transfection Reagent (Thermo Fisher). HEK 293T cells were incubated on six-well plates for 2 days. SDS-PAGE and Western blot was performed on whole-cell extracts by standard methods. The band intensities were captured using a digital image scanner and quantified using Image J (Wayne Rasband, U.S. National Institutes of Health). Primary antibodies used for Western Blots: GFP (Solarbio, RG001030), Phospho-SMAD3 (Cell Signaling, cs 9520, 1:1000), SMAD3 (Cell Signaling 9523, 1:1000), Phospho-p44/42 MAPK (Erk1/2) (Cell Signaling, cs 9101, 1:1000), Phospho-p38 MAPK (Cell signaling, cs 9211, 1:1000), GAPDH (Cell Signaling, cs 2118, 1:1000). Each cell experiment was repeated three times starting from the plasmid transfection procedure. Quantified bands were normalized to housekeeping gene levels (GAPDH). All blots and gels were derived from the same experiment and were processed in parallel.

### Minigene assay

The splicing variants in *MYH3* were experimentally evaluated for potential functional impact on splicing using minigene plasmid pEGFP. The wild-type and mutant plasmids were transfected into HEK293T cells. After incubation for 48 h, total RNA was extracted using the RNeasy Mini Kit (Qiagen, Germany), and cDNA was obtained with the PrimeScript RT reagent kit (Takara, Japan) according to the manufacturer’s instructions. RT-PCR products were examined by agarose gel electrophoresis and Sanger sequencing.

### Patient consent

Written informed consent was obtained in accordance with protocols approved by ethics committee of Peking Union Medical College Hospital (IRB number: JS-908) and from the Regional Ethical Review Board (protocol numbers are 2014/983-31/1 and 2012/2106-31/4). Written consents to use the photographs and radiograms/CT images in this report were obtained from the adult patients and from parents/guardians of all children included in this study. The authors affirm that human research participants provided informed consent for publication of the images in Fig. 2.

### Statistics

SPSS Statistics V22.0 software was used for statistical analyses. Data were analyzed by Student’s *t*-test; the results are shown as the mean ± standard error of *n* = 3 biological replicates. *P* ≤ 0.05 was considered statistically significant.

### Reporting summary

Further information on research design is available in the Nature Research Reporting Summary linked to this article.

## Supplementary information

Reporting Summary

Supplementary information

## Data Availability

The raw sequence data from the Chinese patients have been deposited to the Genome Sequence Archive under accession number HRA001529 (BioProject number PRJCA007177) and can be accessed there. However, according to European law (https://eur-lex.europa.eu/eli/reg/2016/679/oj) the General Data Protection, the raw data from the European patients cannot be shared as a whole data set. Nevertheless, we can share small subsets of variants of interest upon a reasonable request. All variants identified in this study are submitted to ClinVar with the following accession numbers: SCV001572819 to SCV001572827 and SCV001876857.
