# Supplementary material for: Expanding the mutation and phenotype spectrum of MYH3-associated skeletal disorders
Source: NPJ Genom Med. 2022 Feb 15;7:11. doi: 10.1038/s41525-021-00273-x (PMC8847563; doi:10.1038/s41525-021-00273-x)
Supplement: Supplementary file 1 — Reporting Summary [file 41525_2021_273_MOESM1_ESM.pdf]

nature portfolio

Corresponding author(s): Nan Wu
Last updated by author(s): Sep 14, 2021

# Reporting Summary

Nature Portfolio wishes to improve the reproducibility of the work that we publish. This form provides structure for consistency and transparency in reporting. For further information on Nature Portfolio policies, see our Editorial Policies and the Editorial Policy Checklist.

## Statistics

For all statistical analyses, confirm that the following items are present in the figure legend, table legend, main text, or Methods section.

| n/a | Confirmed | |
|---|---|---|
| ☐ | ☒ | The exact sample size (*n*) for each experimental group/condition, given as a discrete number and unit of measurement |
| ☐ | ☒ | A statement on whether measurements were taken from distinct samples or whether the same sample was measured repeatedly |
| ☐ | ☒ | The statistical test(s) used AND whether they are one- or two-sided<br>*Only common tests should be described solely by name; describe more complex techniques in the Methods section.* |
| ☒ | ☐ | A description of all covariates tested |
| ☒ | ☐ | A description of any assumptions or corrections, such as tests of normality and adjustment for multiple comparisons |
| ☐ | ☒ | A full description of the statistical parameters including central tendency (e.g. means) or other basic estimates (e.g. regression coefficient) AND variation (e.g. standard deviation) or associated estimates of uncertainty (e.g. confidence intervals) |
| ☒ | ☐ | For null hypothesis testing, the test statistic (e.g. *F*, *t*, *r*) with confidence intervals, effect sizes, degrees of freedom and *P* value noted<br>*Give P values as exact values whenever suitable.* |
| ☒ | ☐ | For Bayesian analysis, information on the choice of priors and Markov chain Monte Carlo settings |
| ☒ | ☐ | For hierarchical and complex designs, identification of the appropriate level for tests and full reporting of outcomes |
| ☒ | ☐ | Estimates of effect sizes (e.g. Cohen's *d*, Pearson's *r*), indicating how they were calculated |

*Our web collection on statistics for biologists contains articles on many of the points above.*

## Software and code

Policy information about availability of computer code

| | |
|---|---|
| Data collection | The abundance of target relative to control was estimated by densitometry with the ImageJ Software 1.52v. |
| Data analysis | The one-tailed unpaired Student's t test was used for statistical analysis of immunoblotting results. Statistical analyses were performed with SPSS version 21.0 and GraphPad Prism version 8.0.2. |

For manuscripts utilizing custom algorithms or software that are central to the research but not yet described in published literature, software must be made available to editors and reviewers. We strongly encourage code deposition in a community repository (e.g. GitHub). See the Nature Portfolio guidelines for submitting code & software for further information.

## Data

Policy information about availability of data

All manuscripts must include a data availability statement. This statement should provide the following information, where applicable:

- Accession codes, unique identifiers, or web links for publicly available datasets
- A description of any restrictions on data availability
- For clinical datasets or third party data, please ensure that the statement adheres to our policy

The datasets used and/or analyzed during the current study are available from the corresponding authors upon reasonable request.

# Field-specific reporting

Please select the one below that is the best fit for your research. If you are not sure, read the appropriate sections before making your selection.

☒ Life sciences ☐ Behavioural & social sciences ☐ Ecological, evolutionary & environmental sciences

For a reference copy of the document with all sections, see nature.com/documents/nr-reporting-summary-flat.pdf

# Life sciences study design

All studies must disclose on these points even when the disclosure is negative.

| | |
|---|---|
| Sample size | Ten families. |
| Data exclusions | NA |
| Replication | NA |
| Randomization | NA |
| Blinding | NA |

# Reporting for specific materials, systems and methods

We require information from authors about some types of materials, experimental systems and methods used in many studies. Here, indicate whether each material, system or method listed is relevant to your study. If you are not sure if a list item applies to your research, read the appropriate section before selecting a response.

## Materials & experimental systems

| n/a | Involved in the study | |
|---|---|---|
| ☒ | ☐ | Antibodies |
| ☐ | ☒ | Eukaryotic cell lines |
| ☒ | ☐ | Palaeontology and archaeology |
| ☒ | ☐ | Animals and other organisms |
| ☐ | ☒ | Human research participants |
| ☐ | ☒ | Clinical data |
| ☒ | ☐ | Dual use research of concern |

## Methods

| n/a | Involved in the study | |
|---|---|---|
| ☒ | ☐ | ChIP-seq |
| ☒ | ☐ | Flow cytometry |
| ☒ | ☐ | MRI-based neuroimaging |

## Eukaryotic cell lines

Policy information about cell lines

| | |
|---|---|
| Cell line source(s) | A HEK293T cell line was a gift from the Institute of Basic Medical Sciences, Chinese Academy of Medical Sciences |
| Authentication | A HEK293T cell line was a gift from the Institute of Basic Medical Sciences, Chinese Academy of Medical Sciences |
| Mycoplasma contamination | A HEK293T cell line was a gift from the Institute of Basic Medical Sciences, Chinese Academy of Medical Sciences |
| Commonly misidentified lines (See ICLAC register) | None |

## Human research participants

Policy information about studies involving human research participants

| | |
|---|---|
| Population characteristics | We recruited ten families with congenital vertebral malformations and possibly pathogenic variants in MYH3. Families A, B, D and E are of Chinese origin while families C, F, G, H, I and J are of European. |
| Recruitment | Patients with qualifying MYH3 variants were recruited. MYH3 (NM_002470.4) variants were queried from the ES or WGS data and segregation analysis was performed in the families. The variants were prioritized according to expected disease-causing potential, their presence in coding exons ±20 base pairs intronic sequence and a max minor allele frequency of 0.005 according to public (1000G, dbSNP, ExAC/gnomAD, and ClinVar) and two country-specific in-house databases. |
| Ethics oversight | Written informed consent was obtained in accordance with protocols approved by ethics committee of Peking Union Medical |

| Ethics oversight | College Hospital (IRB number: JS-908) and from the Regional Ethical Review Board (protocol numbers are 2014/983-31/1 and 2012/2106-31/4). Written consents to use the photographs and radiograms/CT images in this report were obtained from the adult patients and from parents/guardians of all children included in this study. |
|---|---|

Note that full information on the approval of the study protocol must also be provided in the manuscript.

## Clinical data

Policy information about clinical studies

All manuscripts should comply with the ICMJE guidelines for publication of clinical research and a completed CONSORT checklist must be included with all submissions.

| Clinical trial registration | NA |
|---|---|
| Study protocol | NA |
| Data collection | NA |
| Outcomes | NA |
