# Supplementary material for: Expanding the mutation and phenotype spectrum of MYH3-associated skeletal disorders
Source: NPJ Genom Med. 2022 Feb 15;7:11. doi: 10.1038/s41525-021-00273-x (PMC8847563; doi:10.1038/s41525-021-00273-x)
Supplement: Supplementary file 2 — Supplementary information [file 41525_2021_273_MOESM2_ESM.pdf]

Supplementary Table 1. Summary of the literature reports regarding pathogenic variants (according to NM_02470.4), height and vertebral fusion in *MYH3* related genetic syndromes
Height z-scores reported here were recalculated using original data from the references using https://www.who.int/childgrowth/standards/en/.
NA: not available, mo: months of age, y: years of age, *several affected members from the same family are included

**Freeman-Sheldon syndrome, DA2A**							
Reference	Toydemir et al. 2006	Tajsharghi et al. 2008	Al-Haggar et al. 2010	Beck et al. 2013	Beck et al. 2014	Hague et al. 2015	Ali et al. 2017
Number of affected individuals*	26	1	1	4	15	1	1
Pathogenic variants	c.533C>T, p.(Thr178Ile)<br>c.1493A>G, p.(Glu498Gly)<br>c.1748A>C, p.(Tyr583Ser)<br>c.2014C>T, p.(Arg672Cys)<br>c.2015G>A, p.(Arg672His)<br>c.2474T>A, p.(Val825Asp)	c.533C>T, p.(Thr178Ile)	c.2014C>T, p.(Arg672Cys)	c.737G>C, p.(Gly246Ala)<br>c.1019T>A, p.(Leu340Gln)<br>c.1397T>G, p.(Phe466Cys)<br>c.2503_2505delTTC, p. (Phe835del)	c.533C>T, p.(Thr178Ile)<br>c.1160A>G, p.(Tyr387Cys)<br>c.2014C>T, p.(Arg672Cys)<br>c.2015G>A, p.(Arg672His)	c.2015G>A, p.(Arg672His)	c.2015G>T, p.(Arg672Leu)
Height	NA	Reported to have very short stature at 4y	Reported to have average height at 16mo	NA	NA	NA	NA
Vertebral fusions	NA	NA	NA	NA	NA	NA	NA

**Sheldon-Hall syndrome, DA2B3**					
Reference	Toydemir et al. 2006	Tajsharghi et al. 2008	Alvarado et al. 2011	Beck et al. 2013	Xu et. Al 2018
Number of affected individuals*	12	3	6	4	7
Pathogenic variants	c.533C>T, p.(Thr178Ile)<br>c.782C>T, p.(Ser261Phe)<br>c.875C>G, p.(Ser292Cys)<br>c.1123G>A, p.(Glu375Lys)<br>c.1549G>T, p.(Asp517Tyr)<br>c.2306G>T, p.(Gly769Val)<br>c.2512A>G, p.(Lys838Glu)<br>c.2521_2523delCTC, p.Leu841del<br>c.4865A>C, p.(Asp1622Ala)<br>c.4910C>T, p.(Ala1637Val)	c. 700G>A, p.(Ala234Thr)<br>c.1385A>G, p.(Asp462Gly)	c.1309T>A, p.(Phe437Ile)	c.551G>C, p.(Gly184Ala)<br>c.700G>A, p.(Ala234Thr)<br>c.1512G>T, p.(Lys504Asn)	c.1160A>G, p.(Tyr387Cys)
Height	NA	Two patients with short stature but no numerical values available	NA	NA	Reported to be normal
Vertebral fusions	NA	Scoliosis but vertebral fusions not mentioned	NA	NA	NA

**Contractures, pterygia and spondylocarpotarsal fusion syndrome 1A (CPSF1A)**							
Reference	Chong et al. 2015	Carapito et al. 2016	Zieba et al. 2016	Takagi et al. 2018	Scala et al. 2018	Cameron-Christie et al. 2018	Zhang et al. 2020
Number of affected individuals*	8	4	3	1	1	4	8 (and one fetus)
Pathogenic variants	c.727_729del, p.(Ser243del)<br>c.3214_3216dup, p.(Asn1072dup)<br>c.3224A>C, p.(Gln1075Pro)	c.998C>G, p.(Thr333Arg)<br>c.4031T>C, p.(Leu1344Pro)	c.727-729delTCC, p.(Ser243del)<br>c.1934T>G, p.(Phe645Cys)<br>c. 2699del, p.(Leu900Trpfs*9)	c.5198_5205dup, p.(Met1736fs*10)	c.859T>G, p.(Phe287Val)	c.1986_1990delTTTAA, p.(Asn662Lysfs*15)<br>c.1581+1G>A, splice<br>c.[721A>G;724_725delinsAA], p.(Asn241Asp);(Ser242Asn)	c.3044_3047delinsTCAATTTGTT p.(Glu1015_Asp1016delinsValAsnLeuPhe)
Height	Six patients are reported with short stature, one with normal stature and no data for one patient was available. Numerical values were not provided.	Family 1:<br>II-2: -4.3 at birth, –5,4 adult<br>III-1: -4.3 at 8y7m<br>Family 2:<br>I-2: -0.7 adult<br>II-1: -4.0 at birth	Affected individuals reported to have short stature. Numerical values were not provided.	-3.0 at birth<br>-3.7 at 8y1mo<br>-4.2 at 14y1mo	-1.9 at 8y6mo	NA	Affected family members:<br>-3.4, 72y<br>-3.7, 70y<br>-3.5, 51y<br>-3.1, 29y<br>-3.0, 69y<br>-3.0, 48y<br>-2.8, 46y<br>Unaffected family members (n=4): 0-0,5
Vertebral fusions	Multiple<br>Large intrafamilial variability	Multiple<br>Large intrafamilial variability	Multiple	Multiple<br>Upward protrusion of odontoid causing basilar invagination.	Multiple cervical, thoracal and lumbar partial fusion.<br>Lunotriquetral fusion.	Multiple	Multiple

**Contractures, pterygia,and spondylocarpotarsal fusion syndrome 1B**		
Reference	Cameron-Christie et al. 2018	Hakonen et al. 2020
Number of affected individuals*	6	4
Pathogenic variants	c.4647+1G>A, splice<br>c.-9+1G>A, splice<br><br>c.141T>G, p.(Tyr47*)<br>c.-9+1G>A, splice<br><br>c.1141+131_3256del,<br>(6 kb intragenic deletion beginning within intron 12and continuing to part way through exon 26)<br>c.-9+1G>A, splice	c.1053C>G, p.(Tyr351*)<br>c.-9+1G>A, splice<br><br>c.3102+5G>C, splice<br>c.-9+1G>A, splice
Height	NA	Family A<br>pat 1: -1.1 at birth, -2.8 as adult<br>pat 2: -1.4 at birth, –2.9 adult,<br>Family B:<br>pat 1:-2.3 at birth<br>pat 2: -2.1 at birth.<br>Family B reported to have short stature at 6y and 3y respectively.
Vertebral fusions	Multiple	Multiple

Supplementary Figure 1. Growth of individuals with *MYH3*-associated skeletal fusion syndromes (#618469, #178110) in this and comparison with height/length data from previous studies. Please note variable short stature in patients with both autosomal dominant and autosomal recessive *MYH3*-associated skeletal fusion syndromes.

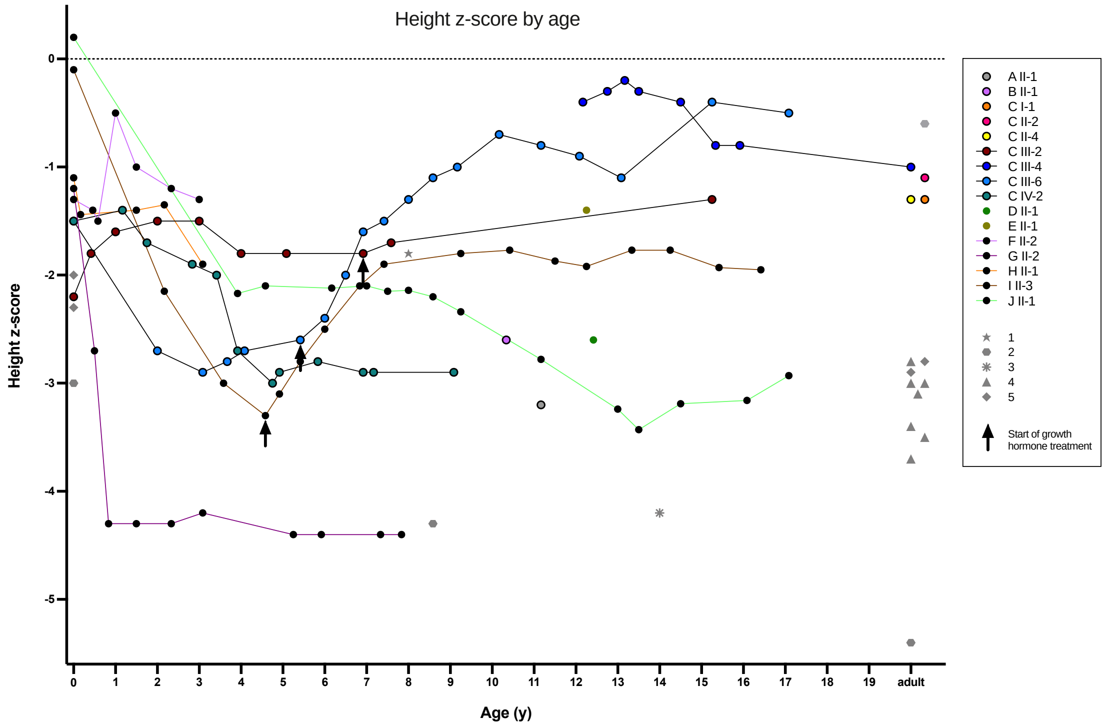

Height data were retrospectively collected from patients and journals and z-scores were calculated using WHO growth standards (https://www.who.int/tools/child-growth-standards/software). Note, that individuals with autosomal dominant and autosomal recessive *MYH3*-associated skeletal fusion syndromes have different and overlapping degrees of short stature. C:III-3, C:III-6 and I:2-1were treated with growth hormone from age of 5 y 6 mo, 7 y and 4 y 8 mo respectively (indicated by black arrows).

Data from the individuals in the study with dominant and recessive MYH3-associated skeletal disorders, is shown using filled-in circles with black boarders and filled-in circles without boarders respectively. Data of the individuals with several data points are joined with a line, whereas data of the individuals where a single data point is available, is depicted only with filled-in symbols. Height z-scores of individuals from previous reports[1-5] are depicted in grey symbols and were calculated from data retrieved from the references listed below and numbered accordingly in grey in the sidebar.

1 Scala, M. *et al.* A novel pathogenic *MYH3* mutation in a child with Sheldon-Hall syndrome and vertebral fusions. *Am J Med Genet A* **176**, 663-667, doi:10.1002/ajmg.a.38593 (2018).

2 Carapito, R. *et al.* Protein-altering *MYH3* variants are associated with a spectrum of phenotypes extending to spondylocarpotarsal synostosis syndrome. *Eur J Hum Genet* **24**, 1746-1751, doi:10.1038/ejhg.2016.84 (2016).

3 Takagi, M. *et al.* A novel truncating mutation in *MYH3* causes spondylocarpotarsal synostosis syndrome with basilar invagination. *J Hum Genet* **63**, 1277-1281, doi:10.1038/s10038-018-0513-0 (2018).

4 Zhang, J. *et al.* Identification of a novel pathogenic variant in the *MYH3* gene in a five-generation family with CPSFS1A (Contractures, Pterygia, and Spondylocarpotarsal Fusion Syndrome 1A). *Mol Genet Genomic Med* **8**, e1440, doi:10.1002/mgg3.1440 (2020).

5 Hakonen, A. H. *et al.* Recessive *MYH3* variants cause "Contractures, pterygia, and variable skeletal fusions syndrome 1B" mimicking Escobar variant multiple pterygium syndrome. *Am J Med Genet A* **182**, 2605-2610, doi:10.1002/ajmg.a.61836 (2020).

Complete author list of the Deciphering Disorders Involving Scoliosis and Comorbidities (DISCO) study

Guixing Qiu[1,2], Zhihong Wu[1,2,3], Terry Jianguo Zhang[1], Nan Wu[1,2], Shengru Wang[1], Jiaqi Liu[4], Sen Liu[1,2], YangYang[1,2], Yuzhi Zuo[1,2], Gang Liu[4], Chenxi Yu[1,2], Lian Liu[1,2], Jiashen Shao[1,2], Sen Zhao[1,2], Zihui Yan[1,2], Hengqiang Zhao[1,2], Yuchen Niu[3], Xiaoxin Li[3], Huizi Wang[3], Congcong Ma[4], Zefu Chen[1,2], Bowen Liu[1,2], Xi Cheng[1,2], Jiachen Lin[1,2], Huakang Du[1,2], Yaqi Li[1,2], Shuang Song[4], Weijie Tian[4], Zhixin Xie[1,2], Zhengye Zhao[1,2], Lina Zhao[1,2], Zhi Zhao[3], Zhifa Zheng[1,2], Yingzhao Huang[1,2]

1. Department of Orthopedic Surgery, State Key Laboratory of Complex Severe and Rare Diseases, Peking Union Medical College Hospital, Peking Union Medical College and Chinese Academy of Medical Sciences, Beijing 100730, China.
2. Beijing Key Laboratory for Genetic Research of Skeletal Deformity, Beijing 100730, China.
3. Medical Research Center, Peking Union Medical College Hospital, Peking Union Medical College and Chinese Academy of Medical Sciences, Beijing 100730, China.
4. Department of Breast Surgical Oncology, National Cancer Center/Cancer Hospital, Chinese Academy of Medical Sciences and Peking Union Medical College, Beijing 100021, China

Full gel image of Westerin blots in Figure 4a

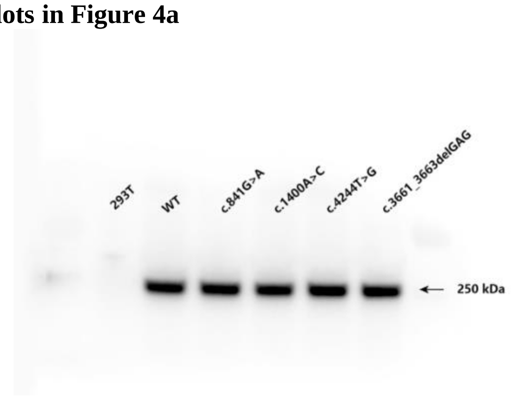

MYH3-EGFP

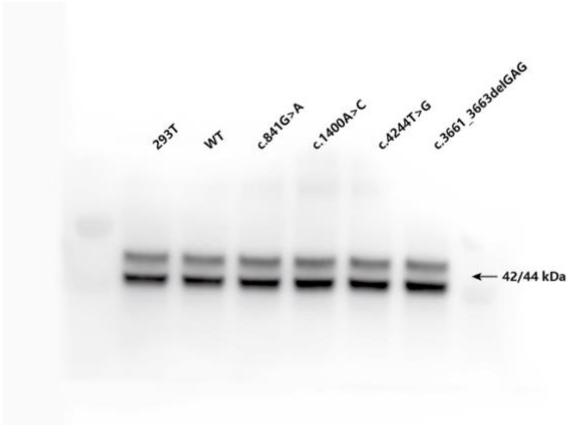

p-ERK1/2

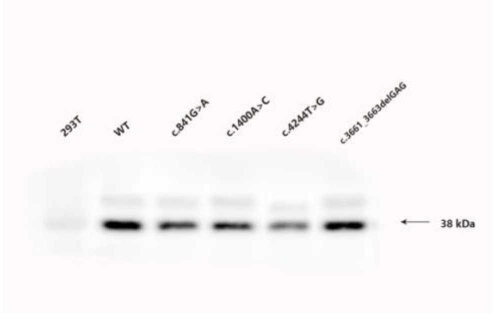

p-p38

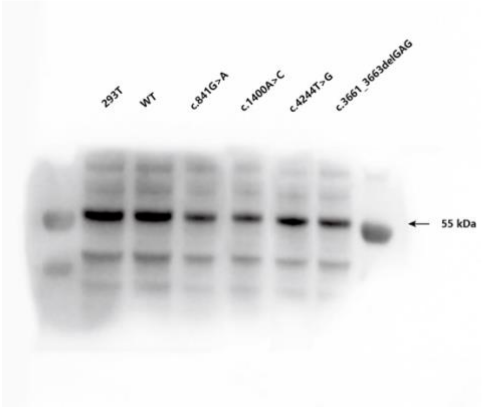

p-SMAD3

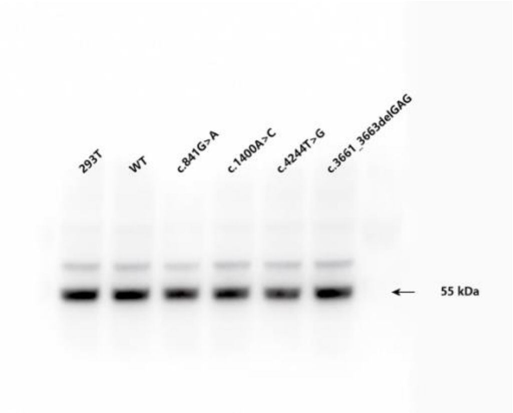
293T
WT
c.841G>A
c.1400A>C
c.4244T>G
c.3661_3663delGAG
55 kDa

SMAD3

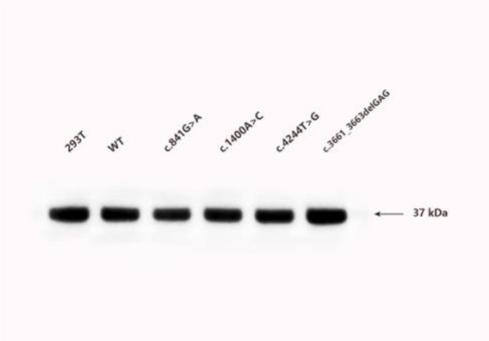
293T
WT
c.841G>A
c.1400A>C
c.4244T>G
c.3661_3663delGAG
37 kDa

GAPDH
